# Calculated Tumor Volume Is an Independent Predictor of Biochemical Recurrence in Patients Who Underwent Retropubic Radical Prostatectomy

**DOI:** 10.1155/2012/204215

**Published:** 2012-05-13

**Authors:** Nobumichi Tanaka, Kiyohide Fujimoto, Akihide Hirayama, Yasushi Nakai, Yoshitomo Chihara, Satoshi Anai, Atsushi Tomioka, Keiji Shimada, Noboru Konishi, Yoshihiko Hirao

**Affiliations:** ^1^Department of Urology, Nara Medical University, 840 Shijo-cho, Kashihara, Nara 634-8522, Japan; ^2^Nara Uro-Oncological Research Group, Kashihara, Japan; ^3^Department of Pathology, Nara Medical University, Nara 634-8522, Japan

## Abstract

*Purpose*. The purpose of this study is to investigate whether the clinicopathological biopsy findings can predict the oncological outcome in patients who undergo radical prostatectomy. *Materials and Methods*. Between January 1997 and March 2006, 255 patients with clinically localized adenocarcinoma of the prostate (clinical T1-3N0M0) who had undergone retropubic radical prostatectomy were enrolled in this study. None of the patients received neoadjuvant or adjuvant therapy. Clinicopathological parameters were assessed to determine a predictive parameter of biochemical recurrence. *Results*. Of the total 255 patients, 77 showed biochemical recurrence during the follow-up period. The estimated 5-year overall survival, 5-year cause-specific survival, and 5-year biochemical recurrence-free survival rates were 97.7%, 99.5%, and 67.3%, respectively. Multivariate analysis using the Cox proportional hazards model showed that calculated cancer volume was an independent predictor among the preoperative clinicopathological parameters (*P* < 0.05). SVI and PSM were independent predictors among the postoperative parameters (SVI; *P* < 0.001, PSM; *P* = 0.049). Among the significant preoperative and postoperative parameters, calculated cancer volume remained an independent predictive parameter in multivariate analysis (*P* < 0.01). *Conclusions*. Tumor volume, as calculated by preoperative parameters, is an independent predictor of biochemical recurrence in patients who had undergone radical prostatectomy.

## 1. Introduction

Radical prostatectomy has been the gold standard of definitive therapy for patients with localized prostate cancer for years [1]. Now, not only laparoscopic radical prostatectomy, but recently also robotic radical prostatectomy has remained popular over the years all across the world [2, 3].

Prostate cancer patients have been able to choose their primary treatment modality among several treatment options since the technical development of radiation therapy such as 3-dimensional conformal radiation therapy (3-DCRT), intensity modulated radiation therapy (IMRT), low-dose-rate brachytherapy (LDR-brachytherapy), and high-dose-rate brachytherapy (HDR-brachytherapy) [4]. If it is possible to predict the oncological outcome based on the clinicopathological findings at diagnosis, it will be possible to choose the most suitable treatment option in patients who receive definitive therapy.

The purpose of this study is to investigate whether the clinicopathological biopsy findings can predict the oncological outcome in patients who are undergoing radical prostatectomy.

## 2. Material and Methods

Between January 1997 and March 2006, there were 577 patients who consecutively underwent retropubic radical prostatectomy at Nara Medical University Hospital and its affiliate hospitals. Among all of these patients, 255 patients who did not receive neoadjuvant or adjuvant therapy and had clinically localized adenocarcinoma of the prostate (clinical T1-3N0M0; 2002 UICC classification [5]) were enrolled in this retrospective study. The mean follow-up period was 53 months (range: 12–127 months). The mean age at surgery and PSA value at diagnosis were 67.4 years and 10.9 ng/mL, respectively.

Patients' course was followed every 3 to 6 months until 5 years after surgery, and then every 6–12 months thereafter. At each visit, PSA was measured and digital rectal examination was performed. If clinical recurrence was suspected, patients underwent a bone scan, computed tomography, and magnetic resonance examination. Biochemical recurrence was defined as a PSA value of 0.2 ng/mL or greater.

Regarding preoperative clinicopathological findings, age at surgery, PSA at diagnosis, biopsy Gleason score, clinical T stage, percent positive biopsy cores, risk classification by D'Amico et al. [6], and calculated cancer volume reported by D'Amico et al. [7] were used to predict biochemical recurrence. Patients were stratified by PSA level at diagnosis of 10 ng/mL or less, greater than 10 ng/mL to 20 ng/mL or less, and greater than 20 ng/mL, respectively, by Gleason score (biopsy and surgical) of 6 or less, 7 and 8–10, respectively. Patients were stratified by a volume of 2.0 mL or less, greater than 2.0 mL to 4.0 mL or less, and greater than 4.0 mL, respectively. Patients were also stratified by positive biopsy core of less than 34%, 34% or greater but less than 50%, and 50% or greater, respectively, and D'Amico risk classification [6] was used for risk classification. Regarding postoperative pathological findings, pathological T stage, extraprostatic extension (EPE), seminal vesicle involvement (SVI), positive surgical margin (PSM), and surgical Gleason score were examined in the same manner.

Biochemical recurrence-free rate was calculated by the Kaplan-Meier method. Comparison of the biochemical recurrence-free rate stratified by clinicopathological parameters was tested by the log rank test. The Cox proportional hazards model was used to determine the predictive parameter of biochemical recurrence among the preoperative and the postoperative parameters both in univariate and multivariate analyses. Finally, the Cox proportional hazards model was used to determine the predictive parameter using the significant predictive parameters among the preoperative and postoperative parameters in multivariate analysis. To examine differences in categorical parameters, the chi-square test was used. Mann-Whitney *U* test was used to examine differences in continuous variables. All *P* values below 0.05 were considered to be statistically significant.

The institutional reviewer board approved this retrospective study, and the obtainment of informed consent from the patients was exempted in view of the aim and methods of this study.

## 3. Results

Of the total 255 patients, 77 showed biochemical recurrence during the follow-up period. Of these 77 patients, 2 patients showed clinical recurrence, and 63 patients underwent salvage therapy (44 patients: androgen deprivation therapy, 11 patients: radiotherapy, and 8 patients: both androgen deprivation therapy and radiotherapy), while 14 patients took a wait-and-see approach after biochemical recurrence. The estimated 5-year overall survival, cause-specific survival and biochemical recurrence-free survival rates were 97.7%, 99.5%, and 67.3%, respectively. The estimated 10-year overall survival, cause-specific survival, and biochemical recurrence-free survival rates were 80.2%, 99.5%, and 56.2%, respectively. Patients' demographics are listed in Tables 1 and 2. PSA level at diagnosis, biopsy Gleason score, clinical T stage, calculated cancer volume, percent positive biopsy core and risk classification were statistically higher in patients who showed biochemical recurrence than in those who did not show biochemical recurrence.

### 3.1. Biochemical Recurrence-Free Rate of Preoperative Clinicopathological Parameters

Regarding the clinical T stage, the estimated 5-year biochemical recurrence-free rates of T1a-b, T1c, T2, and T3a were 80.0%, 74%, 57%, and 51%, respectively. There was a significant difference between T1c and T2 stage (*P* = 0.0379).

 Stratified by the biopsy Gleason score, the estimated 5-year biochemical recurrence-free rates of a Gleason score of 6 or less, 7, and 8–10 were 76.2%, 68.2%, and 24.4%, respectively. Patients with a Gleason score of 6 or less showed a significant higher biochemical recurrence-free rate than those with a Gleason score of 7 and 8–10, respectively (*P* = 0.0377 and *P* < 0.001). There was a significant biochemical recurrence-free rate difference between Gleason score 7 and 8–10 (*P* = 0.0159).

 The estimated 5-year biochemical recurrence-free rates of patients with a PSA level at diagnosis of 10 ng/mL or less, 10.1–20 ng/mL, and greater than 20 ng/mL were 74.4%, 65.7%, and 23.3%, respectively. There were significant differences between the 10 ng/mL or less and the greater than 20 ng/mL groups, and between the 10.1–20 ng/mL and the greater than 20 ng/mL groups, respectively (*P* < 0.0001 and *P* = 0.0002).

 Stratified by the percent positive core, the estimated 5-year biochemical recurrence-free rates of patients with less than 34%, 34% to less than 50% and 50% or greater were 75.3%, 55.0%, and 45.1%, respectively. There were significant differences between patients with less than 34% and those with 50% or greater (*P* < 0.0001).

 Risk classification also showed a significant difference in the biochemical recurrence-free rate. The estimated 5-year biochemical recurrence-free rates of patients with a low risk, an intermediate risk, and a high risk were 79.0%, 71.9% and 48.8%, respectively. The high-risk patient group showed a significantly higher biochemical recurrence rate compared with the low- and intermediate-risk patient groups (*P* = 0.0004 and 0.0375).

 Stratified by calculated cancer volume, the estimated 5-year biochemical recurrence-free rates of patients with 2.0 mL or less, 2.1–4.0 mL, and greater than 4.0 mL were 81.1%, 51.0%, and 12.0%, respectively. Patients with 2.0 mL or less showed a significantly lower biochemical recurrence-free rate than those with 2.1–4.0 mL and greater than 4.0 mL, respectively (*P* = 0.0008, and *P* < 0.0001). Patients with 2.1–4.0 mL also showed a significantly lower biochemical recurrence rate than those with greater than 4.0 mL (*P* = 0.0109).

### 3.2. Biochemical Recurrence-Free Rate of Postoperative Pathological Parameters

Regarding the pathological parameters obtained at surgery, the pathological Gleason score and the pathological T stage were statistically higher in patients who showed biochemical recurrence, and the number of patients who showed EPE, PSM, or SVI was also statistically greater than those without biochemical recurrence.

 The estimated 5-year biochemical recurrence-free rates of pathological T0, T2, T3a, T3b, and T4 were 80.0%, 76.1%, 57.0%, 0%, and 0%, respectively. A log rank test showed significant differences among the pathological T stages.

 Regarding EPE, the estimated 5-year biochemical recurrence-free rates of patients with positive and negative EPE were 72.8% and 53.2%, respectively (*P* = 0.0167). Regarding SVI, the estimated 5-year biochemical recurrence-free rates of patients with positive and negative SVI were 71.0% and 0%, respectively (*P* < 0.0001). Regarding the surgical margin status, the estimated 5-year biochemical recurrence-free rates of patients with a positive and a negative surgical margin were 76.0% and 47.6%, respectively (*P* < 0.0001).

### 3.3. Multivariate Analysis Using Preoperative and Postoperative Clinicopathological Parameters

Regarding the preoperative clinicopathological parameters, biopsy Gleason score, clinical stage, PSA at biopsy, percent positive cores, and calculated cancer volume were independent predictors of biochemical recurrence in univariate analysis. Multivariate analysis using the Cox proportional hazards model showed that the calculated cancer volume was the independent predictor (*P* < 0.05) (Table 3).

Regarding postoperative pathological parameters, surgical Gleason score, EPE, SVI, and PSM were independent predictors of biochemical recurrence in univariate analysis. Multivariate analysis using the Cox proportional hazards model showed that SVI and PSM were the independent predictors (SVI; *P* < 0.001, PSM; *P* = 0.049) (Table 4).

We conducted multivariate analysis using calculated cancer volumes, SVI and PSM, which were significant predictors in multivariate analysis of both preoperative and postoperative parameters. Consequently, SVI and PSM lost their significance and calculated cancer volume was the independent predictor (*P* < 0.01) (Table 5).

## 4. Discussion

Many investigators have tried to determine independent predictors of biochemical recurrence in patients who had undergone radical prostatectomy [8–20]. Among the preoperative clinicopathological parameters, PSA at biopsy, biopsy Gleason score, clinical stage, percent biopsy core, risk classification, and calculated tumor volume were reported as independent predictors, while pathological stage, EPE, SVI, PSM, tumor volume, and surgical Gleason score were reported as independent predictors of biochemical recurrence among the postoperative pathological parameters.

Regarding the predictive potency of tumor volume in biochemical recurrence after radical prostatectomy, some investigators take an affirmative stance [17, 21], while others postulate a dismissive view [22–25]. Taking the affirmative stance, the prediction of the tumor volume of prostate cancer leads to the prediction of biochemical recurrence after radical prostatectomy. This prediction is not only useful in patients who undergo radical prostatectomy, but also those who receive definitive radiation therapy (e.g., IMRT, high-dose-rate and low-dose-rate brachytherapy). An attempt to calculate the tumor volume of prostate cancer has been reported by several investigators [17–21].

In the present study, we calculated the tumor volume according to the equation reported by D'Amico et al. [7]. Using the preoperative parameters, tumor volume had an independent potency of prediction of biochemical recurrence in multivariate analysis. Using the postoperative parameters, SVI and PSM remained as independent predictors. Using these three independent preoperative and postoperative parameters, only tumor volume remained significant. SVI and PSM were marginal predictive values (*P* = 0.073, and 0.058, resp.). On the other hand, Chan and Stamey verified the equation to calculate tumor volume reported by D'Amico, and they reported that there was a significant correlation between the calculated cancer volume and the actual total cancer volume (*r* = 0.537; *P* < 0.0001) [22]. However, they concluded that PSA was a much stronger predictor of cancer volume than calculated prostate cancer volume.

Our present study has several limitations, namely, the number of patients is small (*n* = 255), the mean follow-up period is short (53 months), and we have not calculated the tumor volume by using radical prostatectomy specimens yet. The correlation between calculated tumor volume and true tumor volume in our patients is unknown. However, the calculated tumor volume indeed had an independent predictive potency for biochemical recurrence after radical prostatectomy in multivariate analyses not only among preoperative parameters, but also pre- and postoperative parameters.

 Since the progress in definitive radiation therapy, pretreatment predictive parameters of oncological outcomes after definitive therapy in patients with localized and locally advanced prostate cancer are expected.

## 5. Conclusion

The calculated tumor volume by preoperative parameters can be an independent predictor of recurrence for patients and will experience biochemical recurrence.

## Figures and Tables

**Table 1:** Preoperative clinicopathological parameters.

	All patients (*n* = 255)	Biochem. recur. (+) (*n *= 77)	Biochem. recur. (−) (*n *= 178)	*P* value
Age	67.4 ± 5.8	67.1 ± 5.4	67.6 ± 5.9	n.s.^§^
PSA at biopsy (ng/mL)				
Mean ± SD	10.9 ± 7.2	14.1 ± 9.9	9.6 ± 5.2	<0.001^§^
10 or less	151	35	116	
10–20	84	28	56	
Greater than 20	20	14	6	<0.001*

Biopsy Gleason score				
6 or less	144	32	112	
7	63	20	43	
8–10	25	9	16	
Unknown	23	9	14	<0.001*

Clinical stage				
T1	151	38	113	
T2	102	38	64	
T3	2	1	1	0.001*

Calculated cancer volume (mL)				
2.0 or less	112	17	95	
2.0–4.0	47	17	30	
Greater than 4.0	31	20	11	
unknown	65	23	42	<0.001*
Mean ± SD	2.48 ± 2.38	4.01 ± 3.38	1.88 ± 1.45	<0.001^§^

% positive biopsy core				
Less than 34	158	38	120	
34–50	22	6	16	
50 or greater	66	32	34	
unknown	9	1	8	0.001*

Biochem. recur.: Biochemical recurrence. *Chi-square test and ^§^Mann Whitney *U* test.

**Table 2:** Postoperative clinicopathological parameters.

	All patients (*n* = 255)	Biochem. recur. (+) (*n *= 77)	Biochem. recur. (−) (*n *= 178)	*P* value
Surgical Gleason score				
≦6	122	30	92	
7	89	29	60	
8–10	23	13	10	
Unknown	21	5	16	0.009*

Pathological stage				
T0	5	1	4	
T2	170	40	130	
T3a	68	25	43	
T3b	10	9	1	
T4	2	2	0	0.001*

EPE				
Positive	74	30	44	
Negative	181	47	134	0.017*

Surgical margin				
Positive	64	29	35	
Negative	164	35	129	
Unknown	33	13	20	<0.001*

SVI				
Positive	12	11	1	
Negative	243	66	177	<0.001*

Biochem. recur.: biochemical recurrence, EPE: extraprostatic extension, SVI: seminal vesicle involvement.

*Chi-square test and ^§^Mann Whitney *U* test.

**Table 3:** Univariate and multivariate analysis in preoperative clinicopathological parameters.

Parameter	Univariate			Multivariates		
	Hazard ratio	95% C.I.	*P* value	Hazard ratio	95% C.I.	*P* value
Age (continuous)	0.984	0.944–1.027	0.462			N.A.
Biopsy Gleason score						
6 or less	1			1		
7	1.269	0.663–2.430	0.472	0.658	0.281–1.543	0.336
8–10	4.103	2.199–7.656	<0.001	0.925	0.312–2.739	0.888

Clinical stage						
T1	1			1		
T2	1.719	1.048–2.819	0.032	1.191	0.652–2.177	0.567
T3	4.298	0.583–31.654	0.152	4.037	0.507–32.121	0.187

PSA at biopsy (ng/mL)						
10 or less	1			1		
10–20.0	1.543	0.892–2.668	0.121	0.961	0.398–2.324	0.930
Greater than 20	4.555	2.314–8.965	<0.001	0.892	0.258–3.089	0.857

% positive cores						
Less than 34	1			1		
34–50	1.139	0.444–2.920	0.787	1	0.333–2.999	1.000
50 or greater	2.340	1.384–3.956	0.002	1.807	0.949–3.444	0.072

Calculated cancer volume (mL)						
2.0 or less	1			1		
2.0–4.0	2.716	1.323–5.577	0.006	3.022	1.144–7.981	0.026
Greater than 4.0	7.116	3.595–14.085	<0.001	6.962	1.755–27.624	0.006

N.A.: not available. C.I: confidence interval.

**Table 4:** Univariate and multivariate analysis in postoperative clinicopathological parameters.

Parameter	Univariate			Multivariates		
	Hazard ratio	95% C.I.	*P* value	Hazard ratio	95% C.I.	*P* value
Surgical Gleason score						
6 or less	1			1		
7	1.329	0.748–2.360	0.332	0.884	0.458–1.706	0.713
8–10	3.345	1.716–6.522	<0.001	1.858	0.840–4.113	0.126

EPE						
Negative	1			1		
Positive	2.026	1.232–3.331	0.005	1.337	0.735–2.431	0.342

SVI						
Negative	1			1		
Positive	8.425	4.219–16.825	<0.001	4.615	1.996–10.672	<0.001

Surgical margin						
Negative	1			1		
Positive	2.943	1.704–5.080	<0.001	1.902	1.001–3.614	0.049

EPE: extraprostatic extentions, SVI: seminal vesicle involvement, C.I.: confidence interval.

**Table 5:** Univariate and multivariate analysis in both preoperative and postoperative clinicopathological parameters.

		Univariate			Multivariates		
Parameter		Hazard ratio	95% C.I.	*P* value	Hazard ratio	95% C.I.	*P* value
SVI							
Negative		1			1		
positive		8.425	4.219–16.825	<0.001	2.460	0.919–6.580	0.073

Surgical marrgin							
Negative		1			1		
positive		2.943	1.704–5.080	<0.001	2.057	0.975–4.339	0.058

Calculated cancer volume (mL)							
2.0 or less		1			1		
2.1–4.0		2.716	1.323–5.577	0.006	3.191	1.397–7.291	0.006
Greater than 4.0		7.116	3.595–14.058	<0.001	4.498	1.749–11.564	0.002

SVI: seminal vesicle involvement, C.I: confidence Interval.
